# ABCA1 Transporter Is Involved in the Secretion of CuZn Superoxide Dismutase (SOD)-1 by Activated Human T Lymphocytes

**DOI:** 10.3390/antiox14121487

**Published:** 2025-12-11

**Authors:** Flavia Carriero, Giuliana La Rosa, Luca Pipicelli, Mariarosaria Cammarota, Anna Palmiero, Giovanna Vitolo, Simona Damiano, Mariarosaria Santillo, Francesca Boscia, Giuseppe Terrazzano, Giuseppina Ruggiero, Paolo Mondola, Valentina Rubino

**Affiliations:** 1 Dipartimento di Scienze della Salute, Università della Basilicata, Via dell’Ateneo Lucano 10, 85100 Potenza, Italy; flavia.carriero@unibas.it (F.C.); giuseppe.terrazzano@unibas.it (G.T.); 2 Dipartimento di Medicina Clinica e Chirurgia, Università di Napoli “Federico II”, Via Pansini 5, 80131 Napoli, Italy; giuliana.larosa@unina.it (G.L.R.); luca.pipicelli@unina.it (L.P.); anna.palmiero2@studenti.unina.it (A.P.); simona.damiano@unina.it (S.D.); marsanti@unina.it (M.S.); 3 Dipartimento di Neuroscienze, Scienze Riproduttive ed Odontostomatologiche, Università di Napoli “Federico II”, Via Pansini 5, 80131 Napoli, Italy; mariarosaria.cammarota@unina.it (M.C.); giov.vitolo98@gmail.com (G.V.); boscia@unina.it (F.B.); 4 Dipartimento di Scienze Mediche Traslazionali, Università di Napoli “Federico II”, Via Pansini 5, 80131 Napoli, Italy; valentina.rubino@unina.it

**Keywords:** SOD-1, T lymphocytes, ABCA1, immune activation

## Abstract

The pivotal role of reactive oxygen species (ROS), especially peroxides, in multiple cell signalling pathways has been well-established. Superoxide dismutase 1 (SOD-1) represents a major intracellular source of hydrogen peroxide. Antigen-dependent activation of human T lymphocytes has been previously described by us to induce both SOD-1 production and secretion by T cells. SOD-1 mediated pathways have also been described to deliver proinflammatory signals and to affect the differentiation of immune-suppressor subsets (Treg). The mechanisms underlying extracellular SOD-1 export by activated T cells remain largely undefined. Indeed, SOD-1, like the leaderless proteins, is unable to exploit the conventional trans-Golgi vesicular secretion pathway. Here, we propose that ABCA1 transporters play a role in the mechanisms underlying SOD-1 secretion by activated T cells. Indeed, ABC transporter inhibition by using glyburide significantly decreases SOD-1 secretion by antigen-triggered human T cells in vitro. The effect has been confirmed by using four different detection techniques, as represented by Western blotting, ELISA, flow cytometry and confocal microscopy. Collectively, our findings indicate that ABCA1 transporter-dependent secretion supports the vesicular secretory machinery and might contribute to the extracellular release of SOD-1 by activated T cells. This mechanism highlights ABCA1 as a promising molecular target for therapeutic modulation of deranged immune activation.

## 1. Introduction

The CuZn-containing Superoxide Dismutases (SOD-1 and SOD-3) and the Mn-containing Superoxide Dismutase (SOD-2) isoenzymes represent a large family of antioxidant molecules able to dismutate the O_2_^−^ radical into hydrogen peroxide and molecular oxygen [1,2]. These enzymes play a key physiological role in cellular defence against the reactive oxygen species (ROS) but also participate in fine tuning of multiple intracellular signalling pathways [3,4].

Compelling evidence indicates that SOD-1, which is primarily cytosolic but also detected in traces within the nucleus and mitochondria of all mammalian cells [5,6], is actively secreted analogously to SOD-3 [7] by several cell types [8,9]. Indeed, endogenous mouse SOD-1 has been found to be secreted by the Motor Neuron-Like NSC-34 cells via exosomes [10], while proteomic analysis demonstrated SOD-1 release in rat microglial cells, suggesting a neuroprotective role for this enzyme [11]. The presence of serum SOD-1 [12], mainly associated with low- and high-density lipoproteins, has also been reported in humans [4].

Besides the constitutive time-dependent SOD-1 export, an inducible depolarization-dependent release of SOD-1-containing vesicles has been described in excitable cells and in rat brain synaptosomes. This SOD-1 secretion in response to depolarization involves a vesicular exocytosis pathway mediated by a SNARE-dependent mechanism associated with an increase in intracellular calcium concentration [Ca^2+^] through muscarinic M_1_ receptor-dependent pathways [13,14].

Several mechanisms have been proposed to account for the extracellular export of leaderless proteins, including SOD-1. An unconventional protein secretion (UPS) process, circumventing the canonical Golgi-dependent route and potentially allowing the proteins to translocate into the endoplasmic reticulum (ER) and be directed toward the plasma membrane, has been described [15,16]. Additional mechanisms, such as the translocation through specific membrane pores or the fusion of intracellular vesicles with the plasma membrane, have likewise been proposed [17].

In addition, for potentially cytotoxic polypeptides such as α-synuclein, Tau and other cytosolic misfolded proteins [18,19,20], a misfolding associated protein secretion (MAPS) pathway has been described, characterised by the delivery of proteins to late endosomes, subsequently fusing with the plasma membrane to release their contents. The involvement of this secretion mechanism in the removal of defective proteins that escape proteasomal degradation, due to proteasome insufficiency or dysfunction, has also been proposed [19,21].

A further protein extra-cellular export mechanism is mediated by the ATP-binding cassette (ABC) transporters. These molecules, which perform a wide range of physiological functions [22,23], belong to a large superfamily of membrane proteins, ubiquitously expressed in the eukaryotic cells, that share a common core architecture comprising 12 transmembrane helices [24].

ABC protein superfamily molecules convert the energy derived from the ATP hydrolysis into trans-bilayer substrate movement either into the cytoplasm (import) or out of it (export) [25]. In both processes, ATP hydrolysis is catalysed by a pair of cytoplasmic nucleotide-binding domains (NBDs) [26].

ABC transporters mediate the energy-dependent membrane translocation of a wide variety of substrates including high molecular weight proteins [27]. They are involved in the extrusion of toxic substances and in mediating the multidrug resistance (MDR) phenomenon that frequently occurs during cancer chemotherapy [28,29]. Therefore, drug intra-cellular and tissue compartment trafficking can be extensively limited by ABC transporter availability [30,31].

The involvement of these molecules in regulating cholesterol trafficking within adaptive immune cells [32,33,34], a process consistently associated with antigen-dependent activation of proinflammatory pathways in T cells, has been recently proposed in mouse models [35,36].

Sulfonylurea glyburide (glibenclamide), largely employed in the oral treatment of non-insulin-dependent diabetes, has also been described to inhibit the activity of the ABC-binding cassette transporters in several cells and tissues [37].

The molecular pathways governing SOD-1 secretion under physiological conditions remain largely unknown. We previously showed that antigen-dependent activation of human T lymphocytes acts as a trigger event for both SOD-1 production and secretion [38]. Moreover, SOD-1-mediated pathways have been implicated in delivering proinflammatory signals, as well as in the differentiation of immune-regulatory T cell subsets (Treg) [39].

Recent evidence suggests that ABC transporters play a role in proinflammatory process control; indeed, endogenous substrates including inflammatory mediators (such as prostaglandins, leukotrienes, cytokines, chemokines and bioactive lipids) have been observed to undergo ABC-mediated cell transport pathways [40]. In addition, the dysregulation of these pathways has been widely associated with the pathogenesis/progression of immune-mediated neuronal disorders, such as Multiple Sclerosis [40,41].

Here, to investigate the pathways involved in SOD-1 extracellular export by human immune cells, we analysed the role of the ABCA1 transporter in the mechanisms underlying SOD-1 secretion in antigen-dependent T cell activation.

## 2. Material and Methods

### 2.1. Cells

Peripheral Blood Mononuclear cells (PBMC) were isolated from 10 healthy donors, after informed consent, by centrifugation of peripheral blood on a Ficoll-Paque cushion (GE Healthcare, Uppsala, Sweden) gradient. T cell activation has been carried out using the anti-CD3 mAb (Becton Dickinson, Mountain View, CA, USA) at a concentration of 5 ng/mL. This approach has been widely shown to mimic antigen-dependent T cell stimulation. To investigate the mechanisms involved in both basal and activation-dependent SOD-1 secretion by T lymphocytes, overnight (ON) cultures were set up in RPMI 1640 medium supplemented with 0.2% FBS (Thermo Fisher Scientific, Waltham, MA, USA), alone, in the presence of an activating concentration of the anti-CD3 mAb, as well as with 20 or 30 µM of the ABC transporter inhibitor glyburide (glibenclamide), purchased from Selleck Chemicals, Houston, TX, USA. Cells and culture supernatants, obtained in the above conditions, have been collected as described [38]. Cell viability has been always evaluated by LDH detection in the culture supernatants, as described [38].

Written informed consent (model n. 5526 of Azienda Ospedaliera Universitaria “Federico II”) was obtained from all donors prior to each peripheral blood sampling. Experiments were conducted and analysed anonymously, with no personal information linked to the blood sample. The study was approved by the Institutional Review Board of the Ethics Committee of the University of Naples “Federico II” (Protocol number: 33017, 19 March 2018) and was conducted in accordance with the Declaration of Helsinki, as revised in 2008. All subjects signed their informed consent to the study.

### 2.2. Immunofluorescence and Flow Cytometry Analysis

PBMC were analysed by immunofluorescence using Pe-Cy5 anti-human CD3 (BD Pharmingen, San Diego, CA, USA, clone UCHT1) and anti-SOD-1 mAb (Merk Life Science, Milan, Italy, clone IG6). Intracellular staining was performed with either the BD Cytofix-Cytoperm kit (BD Biosciences, Franklin Lakes, NJ, USA) or the FoxP3 buffer kit (eBioscence, San Diego, CA, USA), following the manufacturer’s instructions. SOD-1 expression in T vs. non-T cells was quantified as the ratio of the mean intensity fluorescence (MIF) in the single-cell subset to the corresponding isotype control mAb, as described [39]. Flow cytometry was carried out on the ATTUNE NxT acoustic focusing cytometer (Life Technologies, Carlsbad, CA, USA). Data analysis was performed by using FlowJo Software (FlowJo, version V10, LLC, Ashland, OR, USA).

### 2.3. Enzyme-Linked Immunosorbent Assay (ELISA)

PBMC were ON cultured in 12-well plates in RPMI medium containing 0.2% FBS, either unstimulated or in the presence of anti-CD3 to induce antigen-dependent activation. Where indicated, cells were treated with glyburide at 20 µM or 30 µM concentration. After incubation, culture supernatants were collected and immediately frozen at −80 °C until analysis. Quantitative detection of human SOD-1 in cell culture supernatants was performed using the commercial ELISA kit (Human SOD ELISA Kit ELK8777, Elk Biotechnology, Sugar Land, TX, USA), according to the manufacturers’ instructions. Optical densities (OD) were measured at 450 nm using an iMark Microplate Absorbance Reader (Bio-Rad Laboratories, Berkeley, CA, USA). Concentrations were calculated by interpolation from the standard curve using nonlinear regression (four-parameter logistic, 4PL) by GraphPad Prism 9 (GraphPad Software, San Diego, CA, USA). The assay detection limit was 1.39 U/L, with an intra-assay coefficient of variation of <8%.

### 2.4. Western Blotting Analysis

Western blotting was performed as previously described [42]. T-lymphocyte intracellular proteins were extracted in RIPA buffer containing 50 mM TrisHCl, pH 7.5, 150 mM NaCl, 1% NP40, 0.5% deoxycholate, 0.1% sodium dodecyl sulphate (SDS), 2.5 mM Napyrophosphate, 1 mM β-glycerophosphate, 1 mM NaVO4, 1 mM NaF, 0.5 mM phenyl-methyl-sulfonyl-fluoride (PMSF), a protease inhibitor cocktail (Roche Applied Bioscience Penzberg, Upper Bavaria, Germany), and a phosphatase inhibitor cocktail (Sigma-Aldrich, St. Louis, MO, USA). Twenty micrograms of intracellular proteins were separated by 10% SDS-PAGE. Extracellular proteins were obtained by placing ON, at 20 °C, 150 µL of culture medium precipitated with 5× of acetone, followed by centrifugation at 15,000 rpm for 20 min at 4 °C. The pellet was resuspended in RIPA buffer and subjected to SDS-PAGE.

Proteins were transferred to a nitrocellulose membrane (GE Healthcare, Amersham PI, UK) using a Trans-Blot Cell (Bio-Rad Laboratories, Berkeley, CA, USA) in transfer buffer (25 mM Tris, 192 mM glycine, 20% methanol). Membranes were blocked in 5% BSA in TBST (tris buffered saline with 0.1% Tween 20; TBST, Bio-Rad Laboratories Segrate Milan Italy) at 4 °C for 18 h and incubated with rabbit polyclonal anti-SOD-1 (ab13498, Abcam, Cambridge, UK) at 4 °C for 18 h. After extensive washing, bands were detected using HRP-conjugated secondary antibodies and chemo-luminescence.

Protein bands were quantified using Image J software (version 1.8, National Institutes of Health, Bethesda, MD, USA). The linearity of the emulsion response was verified using a standard curve of purified SOD-1 run in the same blot. Intracellular SOD-1 was normalised to total ERK immunoreactivity (sc-514302, Santa Cruz Biotechnology, Dallas, TX, USA) while extracellular SOD-1, loaded in equal supernatant volumes, was normalised to the total cellular protein extracted. Protein band analysis was performed by densitometry using Image J software (version 1.8, National Institutes of Health, Bethesda, MD, USA).

### 2.5. Confocal Immunofluorescence Analysis

Confocal immunofluorescence analysis of human T lymphocytes was performed as previously described [43]. Cell were fixed in 4% wt/vol paraformaldehyde in phosphate buffer for 30′ and incubated with the primary antibodies for 24 h: rabbit polyclonal anti-SOD-1 (1:500, ab13498 Abcam, Cambridge, UK); mouse monoclonal anti-SOD-1 (1:400, Merk Life Science, Milan, Italy, clone IG6); mouse monoclonal anti-ABC transporter member A1 (ABCA1; 1:200, SantaCruz Biotechnology, Inc., Dallas, TX, USA) and rabbit monoclonal anti-ABC transporter member A3 (1:400, ab99856 Abcam, Cambridge, UK). Appropriate Alexa488 or Alexa594-conjugated secondary antibodies were used, and nuclei were stained with DAPI. Images were acquired with a Zeiss LSM 700 laser (Carl Zeiss) scanning confocal microscope using a 100× objective, optical sections of 0.7 µm and a resolution of 1024 × 1024 pixels, maintaining identical laser power and exposure for each set. Fluorescence intensity was quantified using the NIH Image, 1.8 version software. The cell surface area occupied by SOD-1 was measured with Image J by thresholding above background and calculating the area fraction.

### 2.6. Statistical Analysis

InStat 3.0 software (GraphPad Software Inc., San Diego, CA, USA), was used to perform statistical evaluation by Mann–Whitney test, Wilcoxon matched-pairs test or Ordinary one-way ANOVA, as indicated. Two-sided *p* values of less than 0.05 were considered to indicate statistical significance.

## 3. Results

### 3.1. Blocking of the ABC Transporters by Using Glyburide Significantly Decreases SOD-1 Secretion by Activated Human T Lymphocytes

To investigate the involvement of ABC transporters in SOD-1 secretory pathways, we analysed the ability of glyburide, an ABC-transporter-blocking drug [44], to interfere with SOD-1 expression and secretion by human T lymphocytes under basal conditions and antigen-dependent T cell activation. The analysis has been performed by using different approaches, as represented by (i) Western blot analysis of SOD-1 intra-cellular and extra-cellular levels in ON PBMC cultures; (ii) ELISA detection of SOD-1 in the culture supernatants; (iii) immune fluorescence and flow cytometry evaluation, in ON cultures, of SOD-1 intracellular levels in activated T cells, as compared with the “non-T” counterpart.

Antigen-dependent T cell activation has been obtained by culturing PBMC with the anti-CD3 monoclonal antibody, able to specifically mimic the physiological antigen triggering of T lymphocytes [38]. The timing and concentration of the different reagents have been defined in preliminary experiments.

**Figure 1:**
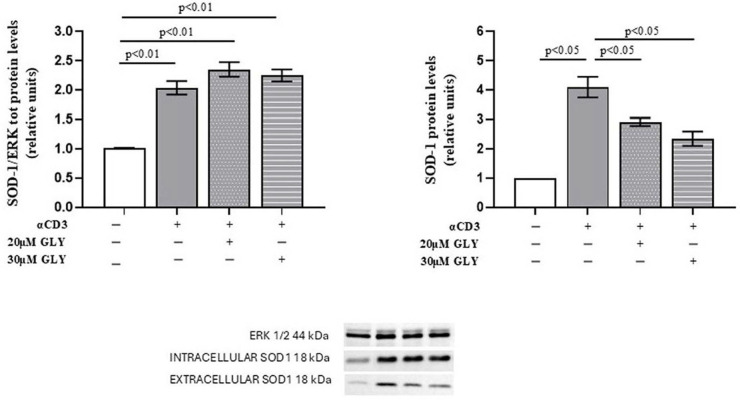
**ABC-transporter-blocking** ** by glyburide treatment** **significantly affects SOD-1** **content of culture supernatants from PBMC ON cultured in the presence of medium or anti-CD3 moAb to mimic antigen-dependent T cell activation.** Western blotting was performed on intracellular and extracellular protein fractions obtained from PBMC cultured ON in medium alone or stimulated with anti-CD3 monoclonal antibody, in the absence or presence of glyburide (20 μM and 30 μM). (**Left**) Densitometric quantification of intracellular SOD-1 normalised to total ERK. A significant increase in intracellular SOD-1 content was observed following T cell activation; glyburide treatment was associated with an increasing trend of intracellular SOD-1 content. (**Right**) Analysis of extracellular SOD-1 from culture supernatants, normalised to total cellular protein content. Activated lymphocytes showed a significant increase in extracellular SOD-1, as compared with the unstimulated cells; as shown, glyburide treatment at both 20 μM and 30 μM concentration significantly reduced the SOD-1 amount. Data are expressed as mean ± SEM in six independent experiments. White, grey, grey-dotted, and grey-striped columns indicate data obtained from supernatants of PBMC cultured with medium, activated with anti-CD3 alone or in the presence of glyburide at 20 μM and 30 μM, as indicated. Statistical analysis was performed by using one-way ANOVA; statistical significance values are indicated.

As shown in Figure 1 (Panel A), Western blot analysis of PBMC, after ON culture in the presence of medium or with the anti-CD3 moAb, was associated with a significant increase in the intracellular SOD-1 content in the activated cells. Glyburide treatment was observed to induce an increasing trend of SOD-1 intracellular levels. When SOD-1 extracellular content in PBMC cultures has been analysed (Figure 1, Panel B), significant increase in SOD-1 was observed in the supernatants of activated lymphocytes; moreover, the presence of glyburide at 20 μM and 30 μM concentration significantly reduced SOD-1 amount in the supernatants of the activated cultures. These results indicate that the pharmacological inhibition of ABC transporters by glyburide markedly decreases SOD-1 extracellular export during antigen-dependent T cell activation. LDH detection was always performed to ensure cell viability, as described [38].

**Figure 2:**
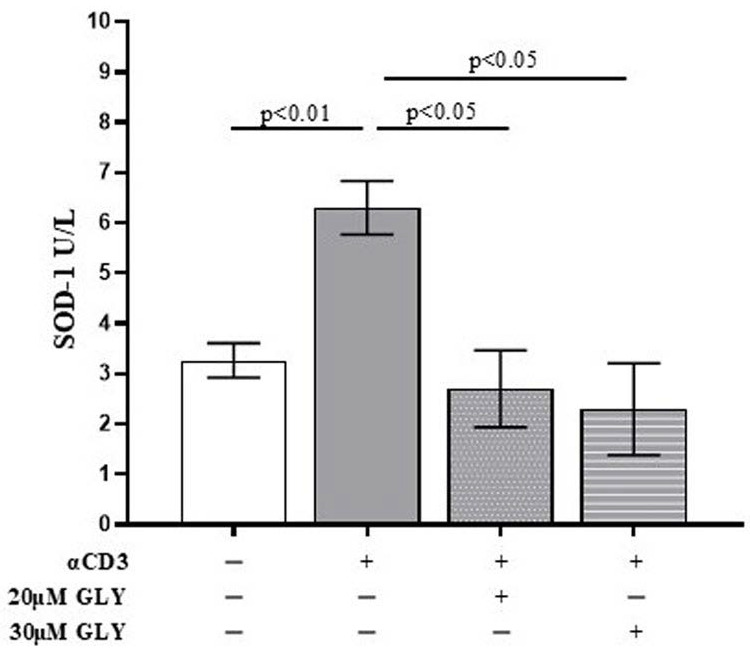
**The quantitative analysis of extracellular SOD-1 content by ELISA detection shows a significant decrease in the enzyme in supernatants from PBMC ON cultured in the presence of anti-CD3 moAb to mimic antigen-dependent T cell activation.** PBMC were cultured ON in the presence of medium, or activated with anti-CD3 monoclonal antibody, in the absence or presence of glyburide (20 μM, 30 μM), as indicated. Culture supernatants were analysed for SOD-1. Data are expressed as mean ± SEM obtained in five independent experiments. White, grey, grey-dotted, and grey-striped columns indicate data obtained from PBMC ON cultured in medium, activated with anti-CD3 alone or in the presence of glyburide at 20 μM and 30 μM, respectively. Statistical analysis has been performed by using Mann–Whitney test; statistical significance values are indicated.

To further investigate the role of ABC transporters in SOD-1 extracellular export by activated T lymphocytes, culture supernatants from ON PBMC cultures were also analysed by ELISA. As shown in Figure 2, supernatants from anti-CD3 stimulated PBMC exhibited a significant increase in SOD-1 levels compared with medium-cultured cells (3.27 ± 0.34 in medium vs. 6.30 ± 0.54 after anti-CD3 stimulation; *p* < 0.01). The addition of glyburide, at 20 μM and 30 μM concentration, markedly reduced extracellular SOD-1 content (6.30 ± 0.54 after anti-CD3 stimulation vs. 2.70 ± 0.76 in the presence of 20 μM glyburide, *p* < 0.05; and 2.30 ± 0.91 in the presence of 30 μM glyburide, *p* < 0.05). No significant effects of glyburide were observed in cultures performed in the presence of medium alone. These results indicate that ABC transporter inhibition significantly impairs SOD-1 export following antigen-dependent T cell activation.

Previous results have been obtained by analysing PBMC cultures as a whole; this strategy, although preserving the heterogeneity of the physiological immunological environment, does not allow the analysis of specific immune cell subsets. Indeed, this heterogeneous cell population includes both T lymphocytes activated through CD3 triggering and a substantial proportion of “non-T” cells, which do not respond to this stimulus and therefore do not undergo the same SOD-1–related changes. In such a mixed cellular context, analysing whole-PBMC lysates may dilute or mask variations occurring specifically within the activated T-cell subset, thereby limiting our ability to detect intracellular SOD-1 changes driven by glyburide treatment.

**Figure 3:**
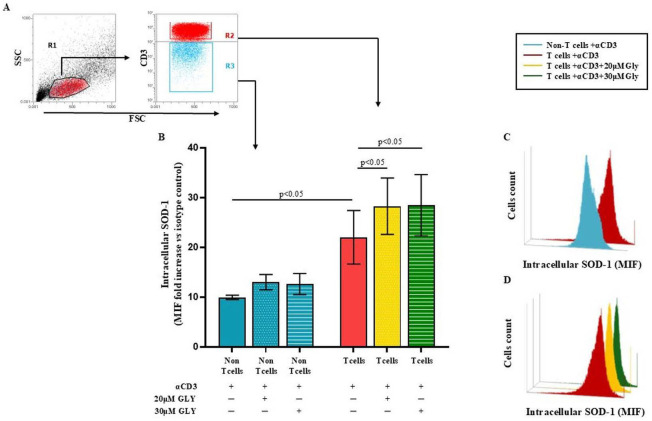
**Flow cytometry analysis of intracellular SOD-1 in T and “non-T” lymphocyte populations following antigen-dependent T cell activation by anti-CD3 triggering, alone or in the presence of glyburide treatment, shows a significant increase in SOD-1 intracellular content only in the T cell subset.** PBMC were cultured ON in medium alone or stimulated with anti-CD3 monoclonal antibody, in the absence or presence of glyburide (20 μM and 30 μM). Intracellular SOD-1 levels were detected by immunofluorescence and flow cytometry. (**A**) Gating strategy to identify the T cell and the “non-T” cell subsets. (**B**) Cumulative data from four independent experiments; SOD-1 content has been shown as the ratio of mean intensity fluorescence (MIF) value for the T and the “non-T” cell subset and the control MIF value obtained after staining the same cell population with the isotype control mAb, as described [39]. Statistical evaluation of data has been performed by means of the Wilcoxon matched-pairs rank test. Statistical significance values are indicated (**C**,**D**). Representative binding profiles showing SOD-1 intracellular amount in T cells after anti-CD3 activation and its further enhancement following glyburide treatment. Data are expressed as mean ± SEM. Light blue, dotted light blue, striped light blue lines represent T cells and “non-T” subset; red, dotted yellow and striped green represent SOD-1 binding in T cells activated in the absence or in the presence of glyburide at 20 μM or 30 μM concentration, respectively. Statistical analysis has been performed by using Wilcoxon matched-pairs rank test; statistical significance values are indicated.

To directly address this issue, we performed additional analyses using immunofluorescence and flow cytometry (Figure 3), which allowed us to selectively examine SOD-1 levels in T cells as well as in the other lymphoid populations (“non-T” cell subsets) unable to be activated by the CD3 triggering. These complementary approaches clearly demonstrate an increase in intracellular SOD-1 exclusively within the activated T-cell population upon glyburide treatment, fully consistent with the expected biological effect.

Panel A of Figure 3 displays the gating strategy employed by us. Panel B of Figure 3 shows cumulative data obtained in four independent experiments by analysing the SOD-1 intracellular level in T and in the “non-T” cell populations in ON cultures performed with medium alone or after addition of the anti-CD3 moAb; as detailed in the Material and Method section, SOD-1 intracellular level has been expressed as the ratio between the mean intensity fluorescence (MIF) value in the T cells and in the “non-T” populations and the control MIF of the isotype control mAb [39]. As shown, anti-CD3 activation significantly increased the SOD-1 intracellular content in the T cells, as compared with the “non-T” cell counterpart (22.02 ± 5.39 in the T cells vs. 9.94 ± 0.44 in the “non-T” cells; *p* < 0.05). Glyburide addition at 20 μM and 30 μM concentration was found to strongly increase the SOD-1 amount in the T cell population (28.27 ± 5.67; with 20μM glyburide addition; *p* < 0.05 and 28.47 ± 6.16 with 30 μM glyburide; *p* < 0.05); glyburide addition does not significantly affect the SOD-1 level in the “non-T” cell population. Panel C and D of Figure 3 show comparative SOD-1 binding fluorescence data in T cells and in the “non-T” cell counterpart in one representative experiment; as displayed, the increasing SOD-1 intracellular level characterises the T cell subset, specifically targeted by the anti-CD3 stimulation (Figure 3C); the ability of glyburide to induce a further SOD-1 intracellular increase in activated T cells has also been shown (Figure 3D).

### 3.2. ABCA1 Transporter Molecules Co-Localise with SOD-1 in the Membrane of Activated Human T Lymphocytes

**Figure 4:**
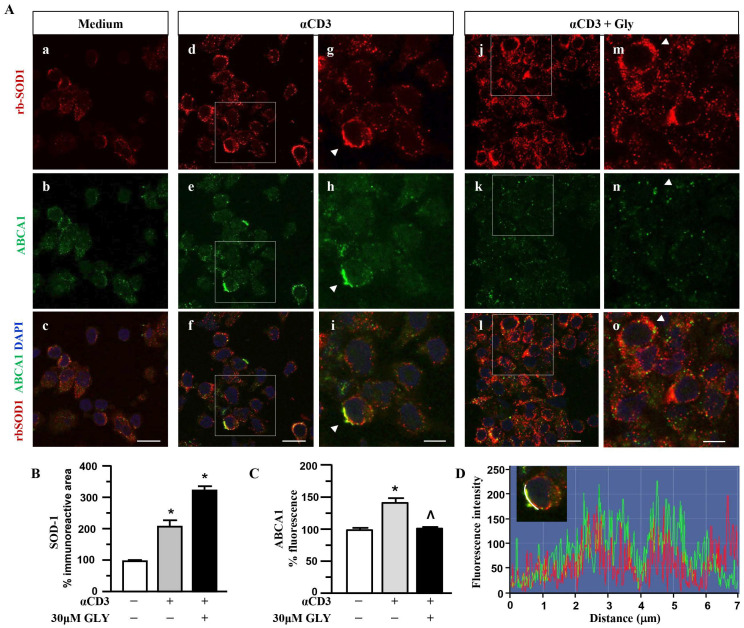
**SOD1 and ABCA1 co-localise in human activated T lymphocytes.** (**A**). Representative confocal images displaying SOD-1 (red) and ABCA1 (green) immunoreactivities in human T lymphocytes ON cultured under control condition (medium, (**a**–**c**)) and after activation with anti-CD3 moAb in the absence (**d**–**i**) or in presence of 30 μM glyburide (**j**–**o**). Panels (**g**–**i**) show high-magnification views of the boxed areas in (**d**–**f**), respectively. Arrowheads in (**g**–**i**) point to cells with a clear SOD-1 immunosignal along the plasma membrane. Arrowheads in (**m**–**o**) point to cells with SOD-1 cytoplasmic immunoreactivity. Scale bars: (**a**–**f**) and (**j**–**l**), 10 μm; (**g**–**i**) and (**m**–**o**), 5 μm. (**B**). Quantitative analysis of SOD-1-immunoreactive area in T lymphocyte cultures under control conditions and after activation, in the absence or in the presence of 30 μM glyburide ON exposure. The values represent the mean ± SEM; (n = 3) * *p* < 0.05 vs. medium. (**C**). Quantitative analysis of ABCA1 fluorescence intensity in T lymphocyte cultures under control conditions, and after activation, in the absence or in the presence of 30 μM glyburide. The values represent the mean ± SEM (n = 3). * *p* < 0.05 vs. medium; ˄ *p* < 0.05 vs. anti-CD3. (**D**). Line profiling of SOD-1 (red) and ABCA1 (green) fluorescence intensities along the line selected on the plasma membrane of anti-CD3 activated T cells.

**Figure 5:**
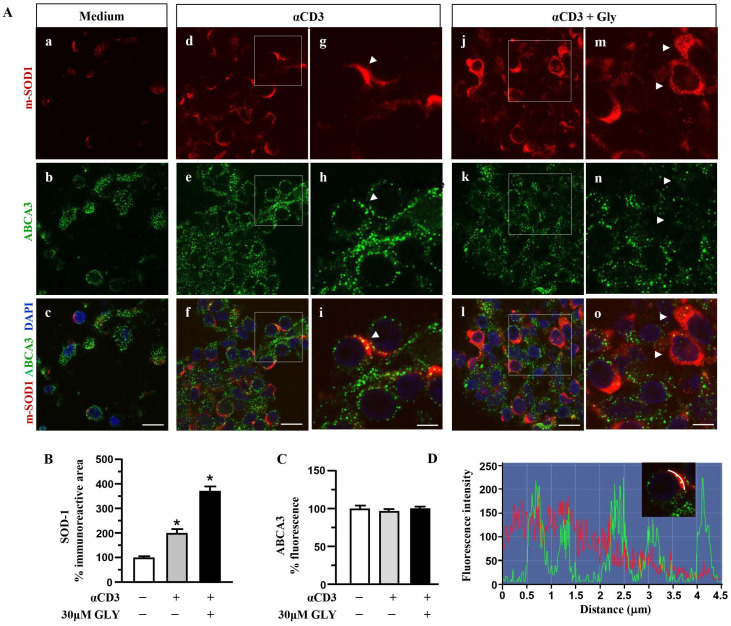
**Distributionanalysis of SOD-1 and ABCA3 in human activated T lymphocytes**. (**A**). Representative confocal images displaying SOD-1 (red) and ABCA3 (green) immunoreactivities in human T lymphocytes under control condition (medium, (**a**–**c**)) and after ON activation with anti-CD3 in the absence (**d**–**i**) or in the presence of 30 μM glyburide (**j**–**o**). Panels (**g**–**i**) show high-magnification views of the boxed areas in (**d**–**f**), respectively. Arrowheads in (**g**–**i**) point to cells with a clear SOD-1 immunosignal along the plasma membrane. Arrows in (**m**–**o**) point to cells with SOD-1 cytoplasmic immunoreactivity. Scale bars: (**a**–**f**) and (**j**–**l**): 10 μm; (**g**–**i**) and (**m**–**o**), 5 μm. (**B**). Quantitative analysis of SOD-1-immunoreactive area in T lymphocytes cultured under control conditions and after activation with anti-CD3 moAb in the absence or in the presence of 30 μM glyburide. The values represent the mean ± SEM (n = 3). * *p* < 0.05 vs. control. (**C**). Quantitative analysis of ABCA3 fluorescence intensity in T lymphocyte cultures under control conditions and after ON activation with anti-CD3, in the absence or in the presence of 30 μM glyburide. The values represent the mean ± SEM. (n = 3). (**D**). Line profiling of SOD-1 (red) and ABCA3 (green) fluorescence intensities along the line selected on the cell plasma membrane of T cells activated with anti-CD3 (**i**) in the absence of 30 μM glyburide.

To further investigate on the involvement of the ABC transporter molecules in SOD-1 secretion by activated T lymphocytes we analysed, by immune fluorescence and confocal microscopy, the cell localisation of SOD-1, ABCA1 (Figure 4) and ABCA3 (Figure 5) family transporter molecules in human T cells. The analysis has been performed in ON cultures in the presence of medium (panels a–c of Figure 4A), of anti-CD3 alone (panels d–i of Figure 4A) or after the addition of the ABC-blocking drug glyburide at 20 μM concentration (panels j–o of Figure 4A).

Panels a–c of Figure 4A show a quite independent distribution of SOD-1 and of the ABCA1 transporter molecules inside the ON medium-cultured human T cells. At variance, anti-CD3 stimulation (Panels d–i of Figure 4A) was observed to significantly increase the intracellular amount of both the SOD-1 as well as the ABCA1 transporter; moreover, the analysis highlights a preferential membrane distribution of both molecules, accompanied by their clear co-localization, confirmed by the quantitative fluorescence overlay analysis showed in Figure 4D. The presence of glyburide in the culture (panels j–o of Figure 4A) strongly affects both SOD-1 as well ABCA1 intracellular amount and distribution; indeed, an increased level of SOD-1 inside T cells, in the presence of a very low content of the ABCA1 transporter, without any clear co-localization of the molecules, might be observed. Such results have been confirmed by the quantitative analysis of the SOD-1 as well as of the ABCA1 transporter (Figure 4C) immunoreactive areas, in different culture conditions. As shown, (Figure 4B) the low amount of SOD-1 in the medium culture significantly increased after anti-CD3 triggering and showed a further rising in the presence of the glyburide treatment; at variance, the ABCA1 intracellular level, augmented by the anti-CD3 triggering, has been observed to revert to the basal amount in the presence of glyburide (Figure 4C). Moreover, glyburide treatment (panels j–o of Figure 4A) strongly hampered the clear SOD-1/ABCA1 co-localization observed in the activated T cells (panels d–i of Figure 4A,D).

When the SOD-1 and ABCA3 family transporter were analysed by immune fluorescence and confocal microscopy (Figure 5), significant upregulation of SOD-1 intracellular immunoreactive area (Figure 5B) was observed in human T lymphocytes after ON culture with the anti-CD3 moAb mimicking antigen-dependent T cell activation. At variance, no significant increase in the ABCA3 intracellular immunoreactive area was measured in the anti-CD3 stimulated T cells (Figure 5C); as shown, (panels d–i of Figure 5A) ABCA3 positive puncta displayed minimal overlap with the intense SOD-1 labelled areas at the plasma membrane sites (panels d–i of Figure 5A,D).

These findings indicate the involvement of the ABC transporters in SOD-1 extracellular export associated with antigen-dependent T cell activation. Moreover, the preferential involvement of the ABCA1 family molecules in this process might also be proposed.

## 4. Discussion

Here we describe that the SOD-1 extracellular export, triggered by antigen-dependent T cell activation, is associated with an ABCA1-transporter-mediated pathway.

The involvement of ROS-mediated signalling in immune regulation has been well-established, while the molecular mechanisms underlying such activity remain largely undefined [45,46,47,48,49]. Moreover, the key role of peroxides, as well as the requirement of a finely tuned control of their availability, amount, timing and intracellular/extracellular location, has been recognised [50,51,52,53].

SOD-1 represents a major hydrogen peroxide intracellular source [3,13]; extracellular export of the enzyme, largely described in eukaryotic cell lines [8,9,54,55], has been found to be associated with specific physiological functions in immune and neurological models [13,14,38,39]. The intracellular and extracellular location of the enzyme in activated T cells strongly support its relevance in the fine regulation of antigen-dependent immune response [4,38,39]. The presence of serum SOD-1, mainly associated with low- and high-density lipoproteins, has also been described in humans, suggesting a protective role of this enzyme on serum lipids [4,12,56].

The relevance of lipid transport for the maintenance of membrane homeostasis in immune effectors has been largely recognised [34,35,36]. Moreover, the fine-tuning of cholesterol availability has been described to be relevant for immune response optimisation [34,35,36]; the mechanisms underlying the calibration of the cellular lipid repertoire in T lymphocytes are largely unknown.

The role of ABCA1 transporter in the control of cholesterol homeostasis in T lymphocytes has been largely demonstrated; recent data propose that Aster-A protein, already described to optimise ABCA1-mediated activity, might regulate proinflammatory T cell function in mouse models [35,36].

Antigen-dependent T cell activation has been found to be associated with rapid SOD-1 intracellular re-localisation and significant induction and extracellular export of the enzyme by activated T cell effectors [38]; moreover, SOD-1 addition to activated T cells in vitro has been observed to enhance their IL-17 production, without significant effects on Interferon (IFN)-γ [39]. These observations support the hypothesis that SOD-1-dependent pathways represent a key player in mediating the homeostatic equilibrium between intracellular/extracellular peroxide availability; such activity might be relevant for the regulation of the complex T_H_1/T_H_17 inflammatory balance.

Multiple mechanisms have been proposed to underlie the extracellular export of SOD-1, as well as of many cytosolic proteins lacking the secretory signal peptide (LLSPs). These molecules have been described to bypass the classical Endoplasmic Reticulum-Golgi pathway (ERGP) by pore-mediated direct translocation across the plasma membrane or via intracellular vesicles fusing with the membrane [16,17,18,19].

A number of cytokines such as IL-1 and IL-18, fibroblast growth factor 2 (FGF2) and redox-enzymes, key players in inflammation and neurodegenerative processes, have been described to also employ ATP-binding cassette transporters in their secretory pathways; moreover, inflammatory mediators, such as prostaglandins, leukotrienes, cytokines, chemokines and bioactive lipids, have been described as ABC transporter substrates [34,40,41]. The involvement of ABCA1-driven processes in the secretion of calpain, a leaderless protein able to modulate IL-17 expression by human T lymphocytes, has also been revealed [54]. Such observations support the potential involvement of ABCA1 transporters in the fine-tuning of immune response. Thus, the relevance of these molecular pathways in pathogenesis/progression of inflammatory brain disorders, such as Multiple Sclerosis, might be hypothesised.

In previous studies [9], we showed that the constitutive SOD-1 secretion in SK-N-BE human neuroblastoma cell line might be inhibited by Brefeldin A, that impairs the classical ERGP, as well as the vesicle pathway export [38]. Similar behaviour has been described by us in activated T cells [38].

Here we show that the mechanisms involved in SOD-1 extracellular export by antigen-triggered human T lymphocytes include ABCA1 transporter contribution. Indeed, ABC transporter inhibition by using glyburide significantly decreases SOD-1 secretion by activated human T cells. This effect has been confirmed by using four different detection techniques, as represented by Western blotting (Figure 1), ELISA (Figure 2), immune fluorescence and flow cytometry (Figure 3) and confocal microscopy (Figure 4 and Figure 5).

Confocal microscopy data indicate the preferential involvement of the ABCA1 family transporter, whose specific functional activity in T cell effectors has been largely described [34]. Moreover, the increased ABCA1 fluorescence levels, likely consistent with the requirement of higher lipid and protein trafficking in activated T lymphocytes, were observed to be modulated by glyburide, suggesting the drug’s inhibitory effect not only on the ABC transporter function, but also on its expression level in activated human T cells.

Taken together, our data indicate that ABCA1 transporter–mediated secretion might support the vesicular pathways for the extracellular export of SOD-1 in activated T lymphocytes [38]. This dual mechanism suggests a coordinated system whereby SOD-1 release can be finely regulated in response to antigenic stimulation, ensuring an appropriate balance of intracellular and extracellular peroxide levels to modulate T cell function and downstream immune signalling. The involvement of ABCA1 transporters is further supported by the presence of a conserved six-residue motif that directs proteins to post-Golgi vesicles, providing a mechanistic link between ABCA1 transporter activity and vesicular trafficking [57].

To the best of our knowledge, this is the first observation highlighting a previously underappreciated role of ABCA1 transporters in immune regulation, extending their known functions beyond lipid and small-molecule transport [34,35,36] to include the control of the secretion of redox-active enzymes, critical for T cell signalling.

Understanding the interplay between ABCA1-dependent and vesicular SOD-1 export pathways may suggest new layers of regulation in T cell activation, cytokine production, and immune homeostasis. Moreover, the dysregulation of these pathways could contribute to the pathogenesis of inflammatory and neuroimmune disorders, such as multiple sclerosis.

## 5. Conclusions

Peroxides have been consistently demonstrated to participate to multiple cell signalling pathways. The involvement of SOD-1, a major intracellular source of hydrogen peroxide, in the fine-tuning of immune response has also been described.

Antigen-dependent T cell activation has been found to induce SOD-1 production and secretion by immune effectors. In addition, extracellular SOD-1 mediated pathways have been described to affect proinflammatory T cell activities in vitro.

Extracellular SOD-1 export mechanisms, by activated T cells, are still largely undefined. Indeed, SOD-1, a leaderless protein, is unable to access the conventional trans-Golgi vesicular secretion pathway.

Here, we propose that ABCA1 transporters participate in SOD-1 secretion by activated T cells. Indeed, by using four different detection techniques, as represented by Western blotting, ELISA, flow cytometry and confocal microscopy, we found that ABC transporter inhibition by using glyburide significantly decreases SOD-1 secretion by antigen-triggered human T cells in vitro.

To the best of our knowledge, this is the first observation highlighting the potential role of ABCA1 in the control of the antigen-dependent secretion of redox-active enzymes, critical for T cell signalling. These findings might represent a fruitful ground for the definition and proposal of new molecular targets to optimise immune-mediated processes in physiological and inflammatory contexts and provide also a framework for future studies exploring the functional consequences of ABCA1-mediated SOD-1 secretion in immune regulation.

### Study Limitations

This study presents several limitations that should be considered when interpreting the findings. The experimental strategy relied predominantly on pharmacological inhibition with glyburide, a broad-spectrum ABC transporter blocker, without the support of genetic loss-of-function approaches (e.g., siRNA or CRISPR/Cas9-mediated ABCA1 silencing). The absence of such complementary strategies limits the ability to ascribe the observed effects exclusively to ABCA1. Moreover, the potential off-target activities of glyburide, particularly its known interactions with additional ABC transporters and ion channels, were not systematically evaluated. Third, the downstream immunological consequences of ABCA1-dependent SOD-1 secretion, including its impact on T cell activation thresholds, cytokine profiles, and effector functions, were not assessed.

The study design included a limited number of experimental conditions and biological replicates, and Western blot analyses were performed on bulk PBMC, potentially masking cell-subset-specific alterations in SOD-1 expression or release.

Finally, this work was intended as a proof-of-concept investigation into the involvement of ABCA1 in SOD-1 secretion. As such, more extensive mechanistic and functional studies, particularly those dissecting cell-intrinsic pathways and immune regulatory networks, will be required to substantiate and expand upon these preliminary observations.

## Data Availability

Data supporting reported results can be obtained by corresponding authors (G.R. and P.M.).
